# Sublingual Buprenorphine/Naloxone and Multi-Modal Management for High-Risk Chronic Pain Patients

**DOI:** 10.3390/jcm10050973

**Published:** 2021-03-02

**Authors:** Shane Kaski, Patrick Marshalek, Jeremy Herschler, Sijin Wen, Wanhong Zheng

**Affiliations:** 1 MS4, West Virginia University School of Medicine, Morgantown, WV 26506, USA; swkaski@mix.wvu.edu; 2 Department of Behavioral Medicine and Psychiatry, West Virginia University School of Medicine, Morgantown, WV 26506, USA; pmarshalek@hsc.wvu.edu (P.M.); jeremy.herschler@hsc.wvu.edu (J.H.); 3 Department of Biostatistics, West Virginia University School of Public Health, Morgantown, WV 26506, USA; siwen@hsc.wvu.edu

**Keywords:** buprenorphine, buprenorphine and naloxone (bup/nal), opioid, pain, substance use disorder

## Abstract

Patients with chronic pain managed with opioid medications are at high risk for opioid overuse or misuse. West Virginia University (WVU) established a High-Risk Pain Clinic to use sublingual buprenorphine/naloxone (bup/nal) plus a multimodal approach to help chronic pain patients with history of Substance Use Disorder (SUD) or aberrant drug-related behavior. The objective of this study was to report overall retention rates and indicators of efficacy in pain control from approximately six years of High-Risk Pain Clinic data. A retrospective chart review was conducted for a total of 78 patients who enrolled in the High-Risk Pain Clinic between 2014 and 2020. Data gathered include psychiatric diagnoses, prescribed medications, pain score, buprenorphine/naloxone dosing, time in clinic, and reason for dismissal. A linear mixed effects model was used to assess the pain score from the Defense and Veterans Pain Rating Scale (DVPRS) and daily bup/nal dose across time. The overall retention of the High-Risk Pain Clinic was 41%. The mean pain score demonstrated a significant downward trend across treatment time (*p* < 0.001), while the opposite trend was seen with buprenorphine dose (*p* < 0.001). With the benefit of six years of observation, this study supports buprenorphine/naloxone as a safe and efficacious component of comprehensive chronic pain treatment in patients with SUD or high-risk of opioid overuse or misuse.

## 1. Introduction

Originally approved by the U.S. Food and Drug Administration (FDA) for out-patient use in 2002, buprenorphine rose to the forefront of the short list of medications for opioid use disorder (MOUD) with the rise of America’s opioid epidemic [1]. Repeatedly, studies have demonstrated the efficacy of buprenorphine therapy in out-patient management of opioid use disorder (OUD) [2,3,4]. Buprenorphine shows equivalent efficacy to the traditional MOUD methadone [5] while possessing a dramatically safer pharmacological profile [6,7]. Additionally, buprenorphine reduces barriers to access with FDA approval for office-based treatment, as compared to methadone which requires that patients travel to a federally approved clinic for their dosing [8].

As one of the hardest-hit states in the nation, West Virginia began developing a comprehensive approach to the treatment of OUD in the early 2000s, focusing on the use of combination buprenorphine/naloxone (bup/nal) as the medication for OUD. This program has since developed into the Comprehensive Opioid Addiction Treatment (COAT) program that treats over 400 patients with eight prescribers spread over three locations [9,10]. The COAT clinic employs a graded approach to group-based therapy and medical appointments, requiring initial weekly contact with a multidisciplinary treatment team that reduces in frequency with increasing duration of sobriety. West Virginia University (WVU) expanded the service in 2014, establishing a High-Risk Pain Clinic to manage chronic pain patients with a history of SUD or otherwise “high risk” behaviors related to opioid or illicit drug use. 

Both pain and SUD are complex and challenging clinical conditions. They are often so intertwined that each can make the other more difficult to treat. A relatively high prevalence of comorbidity has been reported in many previous studies. Even if there is no consistent estimate of an iatrogenic substance use problem, “aberrant medication-taking behaviors” (such as taking more than prescribed or diverting medication to non-prescribed individuals) have become a public health concern [11]. Every day, many people are treated in emergency rooms for misusing or overusing prescription opioids [12]. In primary care settings, one in four people on prescription opioids for noncancer pain struggles with opioid dependence [13]. A systematic review of 38 studies showed an opioid misuse rate of 21–29% and addiction rate of 8–12% in patients with chronic pain [14].

There is no doubt that such patients cannot be appropriately managed with traditional approaches. Integrated treatment models and comprehensive care management have shown promise [15,16,17,18]. Better paradigms incorporating evidence-based MOUD strategies, improving treatment retention, and involving professionally trained mental health clinicians in chronic pain management are worth further study [19]. 

As a partial agonist of μ-Opioid receptor (MOR), buprenorphine has different formulations and is used to treat OUD as well as acute and chronic pain [20]. While the efficacy of buprenorphine compared to traditional opioids in treating pain amongst the general population remains contested [21,22,23], some studies have assessed its use in patients with coexistent SUD, showing an analgesic effect similar to the full agonist methadone [24,25,26]. Given the clinical evidence accumulated from the WV COAT model, buprenorphine makes a plausible choice in managing chronic pain populations with OUD or at risk for opioid misuse or overuse. The present study describes the patient population and outcomes of those enrolled in the WVU High-Risk Pain Clinic.

## 2. Methods

### 2.1. Study Setting

The present study is a retrospective chart review of patients enrolled in the High-Risk Pain Management Clinic of WVU Medicine. The clinic is located in the Pain Management Center of WVU and the patients are managed by a multidisciplinary team comprised of an addiction psychiatrist, a pain psychologist, an addiction counselor, and a case manager. The team works closely with pain specialists and other medical professionals to provide comprehensive pain treatment, including buprenorphine-related products. Because of lower abuse potential, bup/nal film is the first and main option offered to patients unless there is a clear documentation or observed allergic reaction to the combined formulation. The criteria for admission to this clinic include being age 18 and older, chronic pain diagnosis plus SUD, or high-risk opioid use including prescription opioid misuse or overuse, or being identified as high risk by a screening questionnaire such as the Screener and Opioid Assessment for Patients with Pain (SOAPP-R). 

This clinic employs a multimodal approach to pain management centered on the use of buprenorphine for analgesia and includes group meetings, physical therapy, and analgesic injections as well as management of psychiatric comorbidities. Every patient entering treatment received a thorough psychological evaluation in terms of pain and emotional distress. Besides psychiatric treatment for those indicated, the focus of group therapy for all patients in the clinic includes expectation and realistic goal setting, stress management, promotion of a healthy lifestyle, etc. Different techniques such as mindfulness and progressive muscle relaxation are taught and practiced. Healthy activities, hobbies, employment, and education are promoted to help patients build self-esteem, give life meaning, and fend off idle time and boredom that exacerbate pain. Furthermore, patients must agree to regular pill counting and random urine drug screens that must be positive for buprenorphine and its metabolite and negative for any other illicit substance as well as alcohol. The clinic’s highest level of care is biweekly. Treatment is phase-based depending upon treatment adherence such that patients are seen at less frequent intervals from weekly, biweekly, monthly, and every other month as they progress. Patients with SUD diagnosis and abstinence time less than 90 days were first referred to the COAT program for SUD treatment prior to entering the clinic. The High-Risk Clinic has low tolerance for active drug use. At any treatment time, patients may be transferred to the COAT clinic if the treatment team feels focus on SUD is deemed more appropriate. This is because even if the clinic is considered the highest level of ambulatory care in a pain-based setting, there are not enough therapists or supporting staff to manage an acute substance use problem. There was no rapid toxicology screening (only send-out testing) available at the center by the time of this study. 

The study was consistent with protection of patient rights per Health Insurance Portability and Accountability Act (HIPAA) and was approved by the West Virginia University institutional review boards.

### 2.2. Subject Selection

WVU hospitals use an electronic medical record system (EMR) called EPIC for inpatient and ambulatory practice management. We reviewed EPIC charts of all patients who enrolled in the WVU High-Risk Pain Clinic from March 2014 to February 2020. Those who did not remain in the clinic long enough for implementation of standardized pain assessment were excluded.

### 2.3. Pain Assessment 

Pain assessments were conducted at each visit. Level of pain was assessed using the Defense and Veterans Pain Rating Scale (DVPRS). The scale combines a 0–10 pain scale with facial expressions and colors to express pain intensity, with 0 for “No Pain” and 10 for “As bad as it could be, nothing else matters”. The scale also adds supplementary questions to determine the effects of pain on a patient’s daily functions like activity, sleep, mood, and stress. The DVPRS is a self-report pain assessment tool validated in the military population [27]. It is also a preferred pain evaluation tool in other populations because of its friendly and easy-to-use Likert-type scale [28], and inclusion of interference with function and performance [29]. 

### 2.4. Data Collection and Analysis 

Data gathered include demographics, psychiatric diagnoses, prescribed medications, the DVPRS scores (pain and supplementary scores on activity, sleep, mood, and stress), buprenorphine/naloxone dosing, time in clinic, and reason for dismissal. Psychotropic medications comprised prescriptions of Selective Serotonin Reuptake Inhibitors (SSRIs), Serotonin Norepinephrine Reuptake Inhibitors (SNRIs), antipsychotics, mood stabilizers, and other antidepressants such as mirtazapine and bupropion. Pain medications comprised opioids, muscle relaxers, GABA analogues (gabapentin and pregabalin), tricyclic antidepressants (TCAs), anxiolytics/antihistamines, nonsteroidal anti-inflammatory drugs (NSAIDs), and topicals such as lidocaine and capsaicin.

Descriptive statistics were used for patient characteristics. Information analyzed related to treatment included medications utilized prior to admission, medications prescribed by the clinic, buprenorphine total daily dose and dosing frequency, retention rate, and reason for dismissal. Retention rate was calculated as the mean number of days a patient remained enrolled in the High-Risk Pain Clinic. Assessment of the DVPRS pain scales did not begin with the inception of the clinic. To account for this, pain scores were reported relative to number of visits, with the initial assessment of pain score considered as baseline (visit #1). For buprenorphine prescription, one patient was on buprenorphine patch (Butrans) 5 mcg/h; the rest were exclusively prescribed a sublingual bup/nal combination. As we focused on the sublingual bup/nal effect, to avoid skewing data, we did not include this Butrans patient in our calculations involving pain or buprenorphine dosing.

A linear mixed-effects model was used in the longitudinal data analysis on pain score and buprenorphine dose, respectively. This approach can handle fixed and random effect model parameters, as well as nested or unbalanced designs with repeated measures from same subjects. The model included subjects as a random effect and visit number as a fixed effect adjusting for potentially confounding variables such as scores of activity, sleep, mood, and stress.

For visualization, the overall pain assessment for each patient at each visit was aggregated and plotted against the number of visits. Buprenorphine dose at each visit was assessed in the same manner. For the association between pain score and buprenorphine dose, the averaged pain score was plotted at the corresponding dose.

Statistical calculations were performed using Excel 365 (Microsoft Corporation, Redmond, WA, USA) and R software, version 3.6.3 (R Foundation, Vienna, Austria, https://www.r-project.org/). Visualization was through GraphPad Prism v8 (GraphPad Software Inc., San Diego, CA, USA). Outliers were excluded only when identified by the robust regression and outlier removal (ROUT) analysis, which is a unique approach to identifying and removing outliers from nonlinear regression in Prism [30].

## 3. Results

### 3.1. Demographic and Baseline Characteristics

The total number of patients registered in the High-Risk Pain Clinic was 89. Eleven patients were excluded due to having no clinic visit after registration (no standardized pain assessment), leaving 78 total patients included in analysis. The mean age was 53.09 ± 14.46 years (Table 1). Most patients were males (62.8%), and most patients were white (96.2%). The population also had high unemployment and disability rates. Reason for acceptance into the High-Risk Pain Clinic split relatively evenly between prescription opioid misuse/high-risk behavior and SUD.

For psychiatric history, diagnosis of Major Depressive Disorder (MDD) or depression was the most common; anxiety and Generalized Anxiety Disorder (GAD) was the second most common. 

There were 14 different pain diagnoses documented in this population. While chronic back pain was the most common pain diagnosis noted, many patients had more than one type of pain problem.

### 3.2. Psychotropic and Non-Opioid Pain Medication Use

For psychotropic medication use (Table 2), many patients were taking SSRIs (sertraline, paroxetine, citalopram, escitalopram, fluoxetine, and fluvoxamine), SNRIs (desvenlafaxine and duloxetine), and anxiolytic medications or antihistamine sleep aids (alprazolam, diphenhydramine, zolpidem, trazodone, and buspirone) before entering the clinic. Many patients had medication changes during treatment. Large percentages of those who were on SNRIs, mood stabilizers (lamotrigine, lithium, topiramate, valproic acid, and levetiracetam) and antipsychotics (quetiapine, risperidone, olanzapine, and aripiprazole) started them as newly prescribed in the clinic. 

For pain management, 42.3% were prescribed opioid analgesics other than buprenorphine before entering the High-Risk Pain Clinic. The remaining were either obtaining illicit pain pills, buprenorphine, or heroin at least partially for pain control. We also collected information of non-opioid pain medication before and after starting the clinic (Table 2). Many patients were taking NSAIDs, GABA analogues (gabapentin and pregabalin), and muscle relaxants (cyclobenzaprine, methocarbamol, tizanidine, baclofen, and metaxolol) before entering the clinic. For those who were taking SNRIs and tricyclic antidepressants (amitriptyline, dicyclomine, imipramine, doxepin, and nortriptyline), the majority were newly prescribed during treatment time.

### 3.3. Buprenorphine Prescription

**Figure 1:**
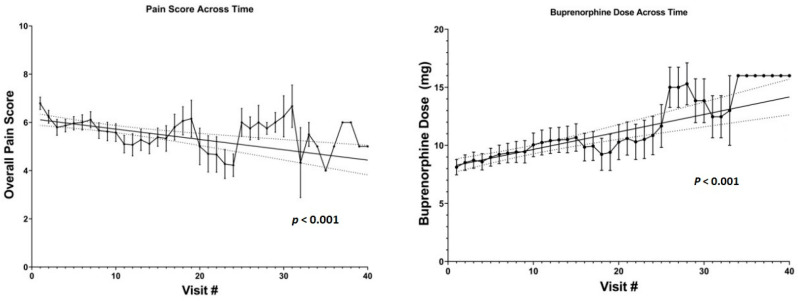
Pain score and buprenorphine dose across time. Data are plotted with mean and error bars.

**Figure 2:**
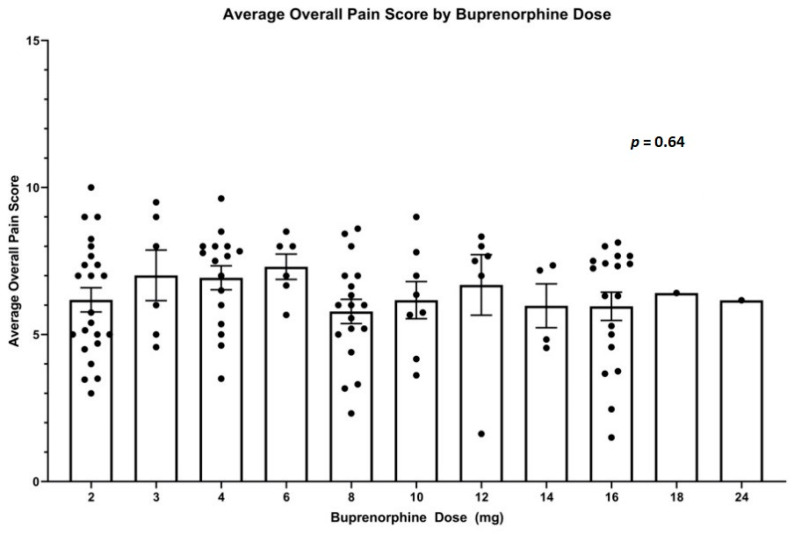
Average overall pain score by buprenorphine dose. Each data point represents the average overall pain score for each subject at the given buprenorphine dose. Data are expressed with mean and error bars.

We found daily dosing of bup/nal was used most commonly (64.3% of prescriptions), followed by twice daily (29.4%), four times daily (5.9%), and three times daily (0.47%). Overall pain scores ranged from 0–10 with a mean of 5.7 ± 2.2. Average overall pain score displayed a downward trend in relation to duration in the High-Risk Pain Clinic (Coeff. = −0.02, *p* < 0.001; Figure 1), while buprenorphine dose showed an upward trend (Coeff. = 0.04; *p* < 0.001; Figure 1). The coefficients (standard error) were estimated from the fixed-effect from the linear mixed-effects model with *p*-values, while the negative (positive) sign implied the decreasing (increasing) trend over time. There was no significant association between overall pain score and the dose of buprenorphine prescribed (*p* = 0.64: Figure 2). In addition, linear mixed model was used to exam the association between the DVPRS Supplementary scores (activity, sleep, mood, and stress) and the visit number. None of them was statistically significant.

### 3.4. Retention

Of 78 total patients analyzed, 21 (26.9%) were referred to a higher level of care and were therefore dismissed from the High-Risk Pain Clinic because of violation of the treatment agreement, mostly with definitive urine drug screen (UDS) results inconsistent with self-reports or evidences of diversion. Twenty-five (32%) left the clinic for reasons unrelated to substance use. For those dismissed due to relapse or inappropriate UDS, five (23.8%) tested positive for unprescribed opioids, four (19%) tested positive for ethanol, four (19%) for THC, three (14.2%) for cocaine, and two (9.5%) for methamphetamine. In addition, two (9.5%) patients had UDS results lacking the prescribed buprenorphine, one (4.8%) did not show for the random buprenorphine count, and one (4.8%) tampered with the UDS. The overall retention was 41% in the five years, 11 months, and three days studied. Mean time until dismissal for relapse or inappropriate UDS was 192.9 ± 114.6 days. Mean time in clinic for non-dismissed patients was 870.5 ± 774.2 days.

Some differences between dismissed and non-dismissed patients are highlighted in Table 3 below. Interestingly, none of dismissed patients carried diagnosis of OUD. The not dismissed group have higher rate of psychiatric comorbidity but a lower average pain score and bup/nal dose. 

## 4. Discussion

We found a downward trend for overall pain score in relation to duration in the High-Risk Clinic, while the average buprenorphine dose showed the opposite trend. The relative high retention rate and long mean time in treatment confirms the feasibility of comprehensive pain and SUD management by multidisciplinary teams. Our study also showed a high percentage of underlying psychiatric illness amongst members of the High-Risk Pain Clinic, particularly depression and anxiety, and as such, a high percentage was prescribed SSRI/SNRI, sleep aids, and anxiolytics. These results are consistent with previous studies demonstrating the increased prevalence of comorbid psychiatric disorders amongst patients with chronic pain [21,31,32], underscoring the importance of mental health professionals in treatment of such a population. 

While some evidence shows benefit to multiple daily dosing of buprenorphine for pain [20], the High-Risk Clinic predominantly utilized daily dosing similar to OUD maintenance therapies, with separation of strips to spread dosing throughout a day discussed on an individual basis with patients. Our analysis found a downward trend for overall pain score but there was no significant relationship between average overall pain score and the dose of buprenorphine, suggesting that some other component of the multimodal clinic may play a significant role in reducing pain scores. The relatively high retention rate demonstrated adds further support for a COAT-adapted integrative care model, as this comprehensive approach may have played an important role in patient retention, and thus pain score improvement, and needs to be investigated further. After all, psychosocial factors including attitudes, beliefs, and mood state all appear to interact with pain and its related behavior [33]. Unfortunately, our analysis did not show significant association of the DVPRS supplementary scores (activity, sleep, mood, and stress) with the treatment time.

While the devastating opioid epidemic highlighted an urgent need for greater caution in opioid prescription, many studies have raised questions about the effectiveness of opioids in the management of chronic non-cancer pain. Systematic reviews revealed that in addition to various side effects that lead to poor retention, opioids in the treatment of most types of chronic pain only show modest rates of improved pain control or patient function [34,35,36]. Moreover, long-term use of opioids can result in hyperalgesia, with exacerbation instead of alleviation of pain [37]. There are also studies demonstrating a strong association between opioid use and poor quality of life as well as functional disturbance [38,39]. The current Centers for Disease Control and Prevention (CDC) Guideline for Prescribing Opioids for Chronic Pain emphasizes that opioids are not first-line for chronic pain and clinicians should evaluate benefits and harms frequently with patients during the course of treatment [40]. This guideline also highlighted the use of buprenorphine and psychosocial intervention for OUD. 

Buprenorphine has an FDA indication for pain [20]. Studies have shown a similar analgesic effect as methadone in patients with SUD [24,25,26]. Long-term use of buprenorphine is safer than full agonists [41,42,43,44], especially in patients who are at a high risk of misuse or overuse. Previous studies on bup/nal therapy in pain patients with opioid dependence found significant decreases in pain [45], improved mood and function [46], and increased retention rate [23]. This is consistent with the findings from the present study.

The WVU High-Risk Clinic makes use of the favorable safety profile of buprenorphine to fill a clinical gap that only increases as more clinicians limit opioid prescriptions. The overall retention rate in our study was 41% and the mean time in clinic for non-dismissed patients was 870.5 days. While opioid drugs constituted 23.8% of all inappropriate UDS dismissals in the present study, ethanol, cocaine, and methamphetamine combined made up 43%, reinforcing the polysubstance nature and the importance of assessment for ethanol and illicit drug use. Furthermore, some differences were noted between patients retained in-clinic and those dismissed. We were surprised to find that 25.6% of patients with a substance use problem were dismissed from the clinic, yet none of them carried the OUD diagnosis. As OUD constituted 28.2% of all patients with substance use history, the increased retention potentially indicates an increased efficacy of the adapted COAT model for that population. 

Interestingly, we noticed this retention rate was higher than that of the COAT clinic, the buprenorphine MOUD program that this clinic is adapted from. In COAT, a sample analysis showed that 37.8% of patients were retained less than one year and 14.7% were retained 10 or more years [9,10]. The increased retention in the High-Risk Pain Clinic may be explained at least in part by a selection effect, as patients with active SUD required at least 90 days of sobriety prior to entering the program. These patients would already be in the intermediate biweekly COAT group. Additionally, the comprehensive approach and focus on functional status and quality of life in addition to OUD may have played a significant role. This may also partially explain why retention was higher in patients with comorbid psychiatric disorder than those with or without SUD.

While the evidence favoring MOUD/replacement therapy to detoxification strategies in OUD is robust [5,47], many current approaches to minimizing prescription opioid use in patients with chronic pain function similarly to detoxification programs. Given emerging similarities in neuroadaptation between these conditions, and their high rate of comorbidity, MOUD/replacement therapy may be a beneficial treatment strategy. This study supports approaching chronic pain in a manner similar to OUD, using medications with safer side effect profiles plus behavioral therapy and psychosocial intervention. Given the fact that 50% of our study subjects have SUD, our results demonstrated that the presence of chronic pain in patients with SUD is not a barrier to meaningful treatment. On the contrary, with a combination of buprenorphine and proper treatment structure, close monitoring, counseling, and psychosocial intervention, patients may stay in treatment longer than with more traditional strategies.

Anecdotally, we have seen patients make remarkable recoveries from pain conditions with buprenorphine that do not respond to other treatment modalities. For example, one patient with complex regional pain syndrome showed a complete recovery with only 4 mg of buprenorphine per day in twice-a-day divided doses. The properties of buprenorphine as mu-opiate receptor partial agonist, producing analgesia, and kappa-opiate receptor antagonist, possibly preventing dysphoria, may provide unique relief of pain and distress [48,49].

This study has four main limitations. First, as with many chart reviews, it was a retrospective study with no control group. Second, even though there were clinical assessments and documentation for most visits, there were not enough data for a good analysis of quality of life and functionality outcomes. Third, we did not have follow-up information for those patients who were dismissed or transferred to a higher level of care such as COAT. These details are very important in any healthcare outcome study especially on a medicine that is commonly used for harm reduction. Finally, the comprehensive and multi-modal nature of the High-Risk Pain Clinic presents a potential confounding factor. Non-pharmacological treatment strategies are employed often in the clinic, though documentation is less rigorous than prescribed medications and therefore difficult to analyze quantitatively. It should also be noted that all patients received continuous addiction psychotherapy and were encouraged to attend 12-step meetings. The treatment agreement included strict rules and options for referral to more comprehensive SUD treatment. All of these may limit the generalizability of the results of the current study.

## 5. Conclusions

SUD and especially OUD complicate the already difficult management of patients with chronic pain. Treating pain and SUD separately is often unsuccessful. The current study presents an example of integrated care that involves multidisciplinary teams and utilizes a stepwise group-based COAT model. Though primarily used in the treatment of OUD, buprenorphine makes a promising substitute for traditional opioids in patients with concomitant chronic pain and SUD/high-risk opioid use behavior. With the benefit of close to six years of observation, the results of the present study support the role of buprenorphine/naloxone as a safe and effective part of comprehensive pain management in these patients.

## Figures and Tables

**Table 1:** Demographic and baseline characteristics.

Age, mean (SD)	53.9 (14.5)
Sex *n* (%)	
Male	49 (62.8%)
Female	29 (37.1%)
Race *n* (%)	
White	75 (96.2%)
Black	3 (3.8%)
Employment *n* (%)	
Employed	34 (43.6%)
Unemployed	38 (48.7%)
Retired	4 (5.1%)
Unknown	2 (2.6%)
Marital Status *n* (%)	
Single	17 (21.8%)
Married	40 (51.2%)
Divorced	21 (27.0%)
Disability *n* (%)	
Yes	36 (46.1%)
No	29 (37.2%)
Unknown	13 (16.7%)
Reason for Admission *n* (%)	
High-Risk Prescription Opioid Use	39 (50.0%)
Substance Use	39 (50.0%)
Substance Use Disorder (SUD)	4 (10.3%)
OUD	11 (28.2%)
Alcohol Use Disorder (AUD)	7 (18.0%)
Cocaine Use	3 (7.7%)
Drug Overdose	2 (5.1%)
Marijuana Use	4 (10.3%)
Street Drug Use	8 (20.5%)
Psychiatric Diagnosis *n* (%) *	
Major Depressive Disorder (MDD)/Depression	40 (51.3%)
Generalized Anxiety Disorder (GAD)/Anxiety	27 (34.6%)
Post-traumatic Stress Disorder (PTSD)	13 (16.6%)
Bipolar Disorder	7 (8.9%)
Somatoform Disorder	5 (6.4%)
Borderline Personality Disorder	2 (2.5%)
Chronic Pain Diagnosis *n* (%) *	
Neck Pain	11 (14.1%)
Shoulder Pain	2 (2.6%)
Hip Pain	3 (3.9%)
Chronic Headache or Migraine	19 (24.4%)
Chronic Lower Back Pain	26 (33.3%)
Spinal Stenosis	14 (18.0%)
Degenerative Disc Disease	6 (7.7%)
Pancreatitis	12 (15.4%)
Osteoarthritis/Arthritis	19 (24.4%)
Osteomyelitis	2 (2.6%)
Abscess	1 (1.3%)
Radiculopathy	4 (5.1%)
Neuropathy	5 (6.4%)
Fibromyalgia	14 (18.0%)

* The percentages do not add up to 100 because of overlap, i.e., patients can have more than one diagnosis.

**Table 2:** Psychotropic and non-opioid pain medication use before and after entering treatment *n* (%).

Medication	Baseline ^1^	In-Clinic ^2^	Newly Prescribed ^3^
Psychotropic Medication Use *n* (%)			
SSRI	25 (32.05%)	28 (35.90%)	7 (25.00%)
SNRI	8 (10.26%)	11 (14.10%)	7 (63.64%)
Antipsychotic	6 (7.69%)	13 (16.67%)	8 (61.54%)
Mood Stabilizer	6 (7.69%)	14 (17.95%)	9 (64.29%)
Anxiolytic/Sleep	16 (20.51%)	23 (29.49%)	10 (43.48%)
Other Antidepressant	5 (6.41%)	9 (11.54%)	4 (44.44%)
Non-opioid Pain Medication *n* (%)			
NSAIDs	45 (57.69%)	57 (73.08%)	32 (56.14%)
SNRI	8 (10.26%)	11 (14.10%)	7 (63.64%)
GABA Analogues	28 (35.90%)	33 (42.31%)	15 (45.45%)
Muscle Relaxant	23 (29.49%)	34 (43.59%)	15 (44.12%)
Tricyclic Antidepressant	7 (8.97%)	19 (24.36%)	14 (73.68%)
Topical	5 (6.41%)	7 (8.97%)	2 (28.57%)

^1^*n* (%) of patients prescribed a given medication before entering clinic. ^2^ *n* (%) of patients that were on a given medication during clinic (continued or newly prescribed). ^3^ *n* (%) of patients from the “In-Clinic” category that were not taking the medication before the clinic. SSRI, selective serotonin reuptake inhibitors; SNRI, serotonin norepinephrine reuptake inhibitors; NSAID, nonsteroidal anti-inflammatory drugs.

**Table 3:** Differences between not-dismissed and dismissed patients.

	Not Dismissed	Dismissed
Reason for Admission *n* (%)		
High Risk Prescription Opioid Use	28 (71.8%)	11 (28.2%)
Substance Use	29 (74.4%)	10 (25.6%)
opioid use disorder (OUD)	11 (100.0%)	0 (0.0%)
Psychiatric Diagnosis *n* (%)		
Major Depressive Disorder (MDD)/Depression	33 (82.5%)	7 (17.5%)
Generalized Anxiety Disorder (GAD)/Anxiety	21 (77.8%)	6 (22.2%)
Post-traumatic Stress Disorder (PTSD)	11 (84.6%)	2 (15.4%)
Bipolar Disorder	4 (57.1%)	3 (42.9%)
Somatoform Disorder	4 (80.0%)	1 (20.0%)
Borderline Personality Disorder	2 (100.0%)	0 (0.0%)
Pain Score and Bup/nal Dose		
Average Pain Score	5.9	7.2
Average Bup/nal Dose (mg)	8.2	8.8

## Data Availability

The data presented in this study are available on request from the corresponding author.
